# Modeling Sustainable Food Systems

**DOI:** 10.1007/s00267-016-0664-8

**Published:** 2016-03-01

**Authors:** Thomas Allen, Paolo Prosperi

**Affiliations:** Bioversity International, Parc Scientifique Agropolis II, 1990 bd de la Lironde, 34397 Montpellier Cedex 5, France; CIHEAM-IAMM, University of Catania, UMR MOISA Montpellier SupAgro, 3191 Route de Mende, 34090 Montpellier, France

**Keywords:** Food and nutrition security, Social-ecological systems, Vulnerability, Resilience, Dynamic systems, Metrics

## Abstract

The processes underlying environmental, economic, and social unsustainability derive in part from the food system. Building sustainable food systems has become a predominating endeavor aiming to redirect our food systems and policies towards better-adjusted goals and improved societal welfare. Food systems are complex social-ecological systems involving multiple interactions between human and natural components. Policy needs to encourage public perception of humanity and nature as interdependent and interacting. The systemic nature of these interdependencies and interactions calls for systems approaches and integrated assessment tools. Identifying and modeling the intrinsic properties of the food system that will ensure its essential outcomes are maintained or enhanced over time and across generations, will help organizations and governmental institutions to track progress towards sustainability, and set policies that encourage positive transformations. This paper proposes a conceptual model that articulates crucial vulnerability and resilience factors to global environmental and socio-economic changes, postulating specific food and nutrition security issues as priority outcomes of food systems. By acknowledging the systemic nature of sustainability, this approach allows consideration of causal factor dynamics. In a stepwise approach, a logical application is schematized for three Mediterranean countries, namely Spain, France, and Italy.

## Introduction

Sustainability has become a guiding principle and a main goal for human development. Environmental degradation, social distress, and economic fluctuation are worldwide concerns challenging conventional views on development and forcing reconsideration of our everyday behaviors. Rapid climate change has been occurring for several decades now and is predicted to continue and possibly accelerate (IPCC 2012). Global biodiversity is declining, with substantial ongoing losses of populations, species, and habitats (UNEP 2012). Increasing land clearance for crop cultivation has been leading to habitat loss and may ultimately result in the loss of plant varieties. Policy needs to strengthen the public perception of humanity and nature as interdependent and interacting. This requires revisiting our policies and behaviors, and developing adaptive management approaches that acknowledge the systemic and dynamic nature of current global changes.

Agriculture and food systems are at the center of debates over sustainability. The processes underlying environmental, economic, and social unsustainability derive in part from the global food system. Significant trade-offs have accompanied the increase in food supply. Processes along the food chain from agricultural production to food consumption produce outputs other than consumable food that are returned to the natural environment such as pollution or waste. Food waste alone represents around 3–5 % of global warming impacts, more than 20 % of biodiversity pressure, and 30 % of all of the world’s agricultural land (EU 2014). Meanwhile, 842 million people still suffer from undernourishment (FAO 2013), while obesity has become a significant public health issue with 500 million obese adults (Finucane et al. 2011). Building sustainable food systems has become a popular motto and a major endeavor to redirect our food systems and policies towards better-adjusted goals and improved societal welfare.

A sustainable food system can be defined as one that “provides healthy food to meet current food needs while maintaining healthy ecosystems that can also provide food for generations to come, with minimal negative impact to the environment; encourages local production and distribution infrastructures; makes nutritious food available, accessible, and affordable to all; is humane and just, protecting farmers and other workers, consumers, and communities” (Story et al. 2009). The food system has a high level of complexity driven by many economic, socio-cultural, and environmental factors, which are both internal and external to its boundaries. The systemic nature of these interactions calls for systems approaches and integrated assessment tools to guide change.

Many intricately related factors are involved in getting food from farm to consumer, including the inputs, processes, and outcomes of food systems. Food systems act as complex social-ecological systems, involving multiple interactions between human and natural components. Better understanding of these drivers and how they interact to influence activities and outcomes of the food system can help improve public policies. Efforts to define, measure, and model progress towards sustainability have led to the development of a variety of indicators and models that monitor and simulate (some) aspects of sustainability. In this paper, we present an additional approach that considers vulnerability and resilience as the operating concepts to model the systemic factors that lead to final food system outcomes such as food and nutrition security.

Food and nutrition security remains a crucial policy issue in every country, and the current global crisis of malnutrition is an urgent concern for both developed and developing countries. The proponents of the “Sustainable Diet” agenda, a closely related concept highlighting the role of consumers in defining sustainable options, provide in particular a food and nutrition security-orientated perspective on the question of the sustainability of food systems (FAO/Bioversity 2012; Johnson et al. 2014). Transforming the abstract concept of sustainability into descriptive objectives, the authors propose in this paper a conceptual model that articulates crucial vulnerability and resilience factors to global environmental and socio-economic changes in the Mediterranean region, postulating specific food and nutrition security issues as priority food system outcomes. Identifying and modeling the intrinsic properties of the food system that will ensure that its essential outcomes are maintained or enhanced over time and across generations can help organizations and governmental institutions track progress towards sustainability and set policies that will encourage positive transformations. The Latin Arc countries—Spain, France, and Italy^1^—have been selected as the study area due to the biophysical and socio-economic common features of this transnational area.

The first section of the paper reviews the background and theory of sustainability, recalling that assessment exercises aim at identifying fundamental systemic properties. We discuss, in particular, the concepts of vulnerability and resilience proposed in social-ecological system frameworks as key concepts for sustainability assessment. Building on dynamic system theory, we then suggest a formal representation of the overall food system to structure its different elements; clarify the distinctions between input, state, and output variables; and formalize the scale at which system dynamics are operating. In the third section, we present a stepwise application of the model, identifying specific drivers and issues for the Latin Arc and formulating explicit interactions. We finally motivate this approach in the Discussion section.

## Identifying the Fundamental Sustainability Properties of the Food System

### Sustainability as a System Property

The multidimensional nature of sustainable development—which has to satisfy several economic development, social equity, and environmental protection goals—is generally emphasized. Proponents of sustainable agriculture have for instance proposed alternative farming practices, which are less environmentally impacting but also embedded in new sets of values and carrying other visions of organization in society. These renewed approaches to agriculture—such as organic farming, low-input agriculture, biodynamic agriculture, regenerative agriculture, permaculture, and agroecology—are interesting crucial initiatives rooted in the ground. Yet, sustainability in agriculture cannot be defined per se by the simple adherence to one of these approaches; these are propositions of solutions towards sustainability.

The most frequently quoted definition of sustainability comes from *Our Common Future*, also known as the Brundtland Report (UN 1987). Human development must meet “the needs of the present without compromising the ability of future generations to meet their own needs.” This forward-looking imperative highlights the inter-generational and inter-temporal dimensions of sustainability, which thus *infer* that stewardship of both natural and human resources is of prime importance to ensure long-term development. When applied to the agricultural and food sector, Conway’s frequently quoted definition of agro-ecosystem sustainability refers to “the ability of a system to maintain productivity in spite of a major disturbance, such as caused by intensive stress or a large perturbation” (Conway 1985). Hansen (1996) further interprets sustainability as a system’s ability to continue over time. The concept of agricultural and food sustainability refers to a property of a system, rather than an approach to agriculture. Only such an understanding can offer a way out of the logical flaw of judging the sustainability of approaches that have been defined in the first place as sustainable, and allow the assessment of the contribution and suitability of these approaches towards sustainability.

Sustainability is a property of a system that is open to interactions with the external. It is the dynamic preservation, over time, of the intrinsic identity of the system among perpetual changes (Gallopín 2003). Multiple factors influence the course of human–environment interactions, which are further complicated by the presence of co-evolving causal forces. Research in both the natural and social sciences uses the idea of a system to explain complex dynamics. A system is a network of multiple variables that are interconnected through causal relationships. Modern societies depend on complex systems to provide food (Fraser et al. 2005). Food systems encompass an array of activities from sowing through to waste disposal management, including production, processing, packaging and distributing, and retail and consumption (Ericksen et al. 2009). Furthermore, global environmental and socio-economic changes are occurring concurrently, affecting food activities. Food systems, in turn, have an impact on the environment as activities and outcomes are also drivers of global environmental change, engendering feedback loops and cross-scale interactions. If assessing sustainability is about understanding these dynamics to gauge the ability of a system to maintain or enhance its essential outcomes, viewing the system as a whole is essential. Systems thinking can be a useful approach to capture causal loops, where the effects of the last element influence the input of the first element. The coupled Human–Environment System or the Socio-Ecological System (SES) (Holling 1996; Turner et al. 2003; Ericksen 2008; Ostrom 2009) approaches allow us to move away from focusing solely on isolated events and their causes, and to look at systems made up of interacting parts. The analysis and assessment of the sustainability of the food system are here conducted through the application of an SES framework.

### A Social-Ecological Framework

SES frameworks originate from ecosystem management and ecology. SESs can be defined as complex human–nature adaptive systems linked by dynamic processes and reciprocal feedback mechanisms, with a substantial exchange of energy and materials across boundaries (Berkes et al. 2001; Folke 2006). A crucial challenge towards sustainability of food systems is the management of dynamics originating from both global and internal changes, and their different synergistic impacts on systems’ outcomes. Only a better understanding of these processes will help us estimate and forecast trade-offs between human wellbeing and ecosystem services, economic performances, and environmental impacts. Vulnerability and resilience have emerged in recent years as key SES framing concepts for research on global change (Downing 2000; O’Brien and Leichenko 2000; McCarthy et al. 2001; Turner et al. 2003; Schröter et al. 2005; Polsky et al. 2007). Vulnerability/resilience assessment and modeling are today acknowledged methods to explore sustainability of SES. There are several illustrations of approaches analyzing food systems for their vulnerability and resilience to global socio-economic and biophysical changes in order to explore sustainability, and highlighting key system processes and characteristics (Ericksen 2008; Darnhofer et al. 2010; Allouche 2011).

Vulnerability and resilience constitute differing yet overlapping research themes (Turner 2010). Both address the consequences and the responses of a system to social and/or environmental changes. The differences in their respective approaches to social-ecological dimensions of change remain in discussion (Miller et al. 2010). For a comprehensive review, see Alwang et al. (2001). Ericksen (2008) argues that the vulnerability approach “frames the consequences of environmental change for food systems in the context of socioeconomic and political change so as to understand the synergistic effects of the multiple stresses that interact with food systems, sometimes making these systems vulnerable.” A common thread of (almost) all approaches to vulnerability is the consideration that it is an “intrinsic characteristic of a system” that is at risk. The conditions and properties of the exposed system—or element of the system—are the crucial features to be identified and assessed (Birkmann 2006). In the meantime, vulnerability deals also with features linked to capacities of the system to anticipate and cope with the impact of a change or hazard (Bohle 2001). This allows flexibility in applying vulnerability for largely different elements, such as structures and physical characteristics of buildings, ecosystems, and environmental functions and services, but also communities and social groups.

The concept of resilience, originating in ecology, is central to visualizing the dynamics of the coupled system. Resilience is interpreted differently by SES scholars but commonly recognized as a multi-attribute concept composed of (i) an ability to cope with disturbance or change and retain control of function and structure; (ii) a capacity to self-organize; and (iii) a capacity to learn and adapt (Walker et al. 2002; Berkes et al. 2003; Walker et al. 2004). Both vulnerability and resilience assessment highlight the need for methods and metrics that do not simply express final results or outcomes, but provide a system of information that can be interpreted in a causal framework, modeling interactions between different variables.

Building on Turner et al. (2003), the conceptualization of sustainability as the dynamic ability of a given system to maintain or enhance its essential outcomes over time allows vulnerability and resilience theories to provide the elements to understand the mechanisms likely to affect activities within the system. The challenge for SES framework analysis here is to identify the pathways leading to vulnerability, and the characteristics and opportunities ensuring resilience of the food system in a context of change. Since contemporary food systems are characterized by cross-scale interactions and feedbacks across time and space as well as between social and ecological components (Cash and Adger 2006), efforts to rate how changes affect the performance of social, ecological, and economic systems over time are crucial for progress to be made towards sustainable development (Gallopín 2003). At the same time, desired systemic properties can be expanded by investing in specific components of systems (Marschke and Berkes 2006). In particular, the vulnerability framework can be disaggregated in several dimensions according to different drivers of change: vulnerability to climate change, vulnerability to price volatility, vulnerability to demographic transformations, etc.

### Vulnerability/Resilience for the Analysis of Food System Sustainability

Vulnerability in SES depends on the stress to which a system is exposed, its sensitivity, and its adaptive capacity and resilience opportunities. In line with the internationally recognized IPCC definition, De Lange et al. (2010) state that “Vulnerability is generally considered as a function of exposure to a stressor, effect (also termed sensitivity or potential impact) and recovery potential (also termed resilience or adaptive capacity).” This definition proposes a clear and synthetic definition of vulnerability in terms of its components that are fundamental for the modeling exercise. *Exposure* refers to the existence or presence of elements^2^ in the system that are susceptible to be adversely affected by the occurrence of environmental or socio-political stresses (IPCC 2012). It is a necessary but not sufficient first condition for a given system to experience stress or perturbations. *Sensitivity* is the degree to which a system is potentially affected by its exposure to a stress or perturbation (Adger 2006). It can be understood as the potential magnitudes of consequences of being exposed (Downing 1991). Indicators of sensitivity measure generally impacts. See Prosperi et al. (2014) for further clarification.

*Recovery potential* is composed of adaptive capacities and resilience opportunities. These are related to the potential of a system to respond to changes, including adaptation and transformation (IPCC 2001; Burton et al. 2002; Adger et al. 2003). Adaptation captures the capacity of a system to learn and adjust to changing processes, and “continue developing within the current stability domain or basin of attraction” (Berkes et al. 2004, cited in Folke et al. 2010). Systems with high recovery potential will absorb disturbances and retain their original structure and processes. Transformation has been defined as “the capacity to create a fundamentally new system when ecological, economic, or social structures make the existing system untenable” (Walker et al. 2004). Transformation is then necessary for the system to maintain its functionalities. Resilience is more specifically concerned with the ability of a system to “absorb shocks, to avoid crossing a threshold into an alternate and possibly irreversible new state, and to regenerate after disturbance” (Resilience Alliance 2010). Resilience is the ability of a system and its component parts to anticipate, absorb, accommodate, or recover from the effects of a hazardous event in a timely and efficient manner by ensuring the preservation, restoration, or improvement of its essential basic structures and functions.

**Fig. 1:**
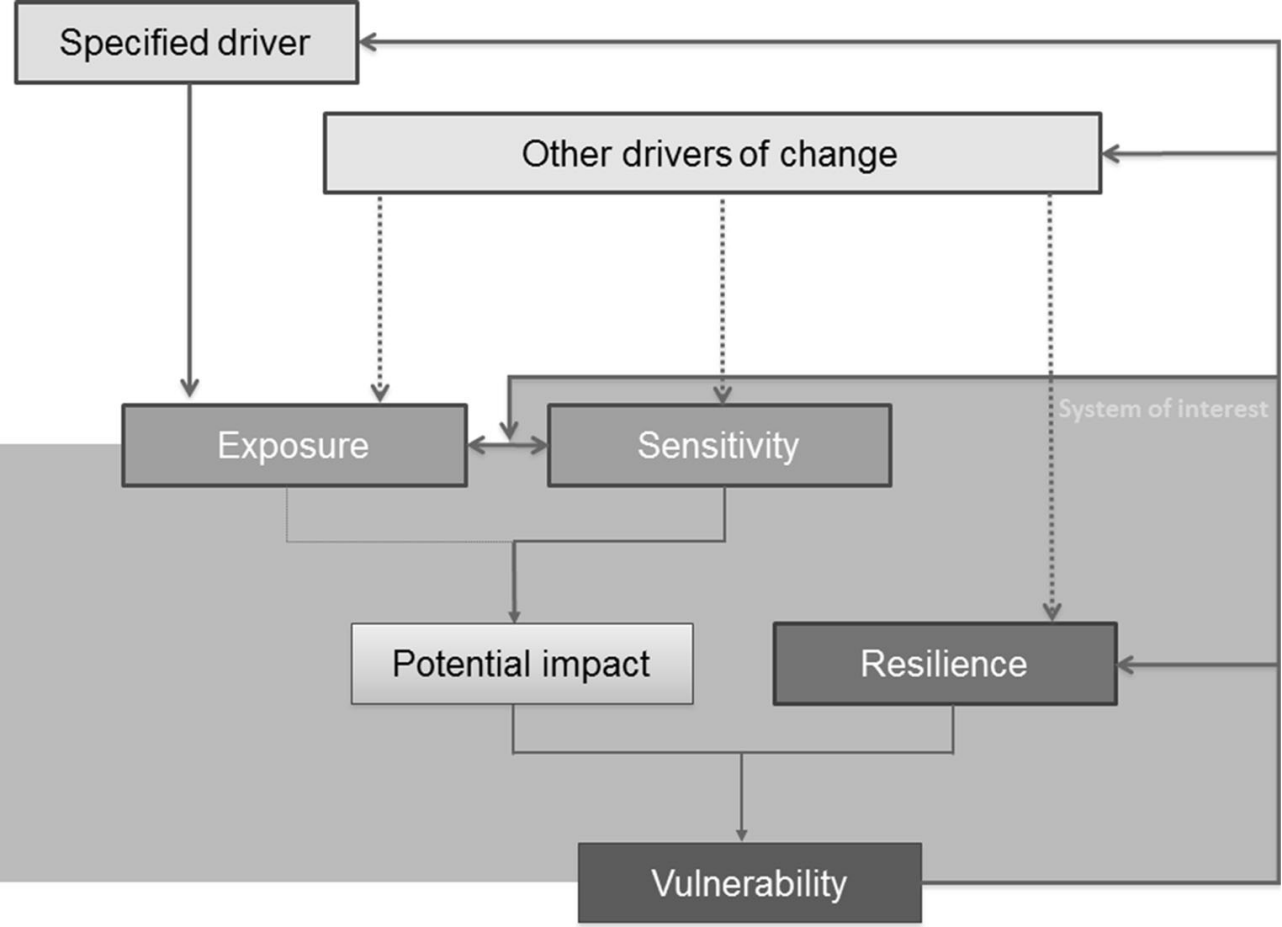
A causal pathway (adapted from Fussel and Klein 2006)

Exposure refers to relational variables, i.e., elements that characterize the relationship between the system and its environment (Gallopín 2006). It is the first point of contact between the stress or perturbation, and the system. Although commonly included in vulnerability (Chambers 1989; Adger and Kelly 1999; IPCC 2001; Turner et al. 2003; Polsky and Eakin 2011), exposure has recently been excluded from vulnerability in the last IPCC definition to actually align the understanding of vulnerability as a pure attribute of a system existing prior to and apart from the disturbance. In the earlier IPCC definitions,^3^ reference was indeed made as well to information on the change itself (e.g., its magnitude, rate of variation, duration, etc.), as well as on the presence of elements that are exposed. The question of whether vulnerability is determined purely by the internal characteristics of a system, or whether it also depends on the likelihood that a system will encounter a particular hazard, is the subject of a long-standing dispute (Brooks 2003). We will consider here the conventional framework for vulnerability. The understanding of exposure as the first interface with a specific driver of change helps differentiating it from the sensitivity or resilience components, which might be influenced by other drivers of change (Fig. 1) (Fussel and Klein 2006).

When a food system fails to deliver food security or has the potential to do so in the face of a perturbation, the system can be considered as vulnerable (Ericksen 2008). Foran et al. (2014) state that “The social-ecological system considers the human-environment interface as a coupled ‘system’ where socio-economic and biophysical drivers of change interact to influence activities and outcomes, of the food system, that subsequently influence drivers of changes in a feedback loops dynamic.” Such systems can exhibit coherent behaviors. Constituting elements interact in a complex but reasonably lawful way. How can we account for the confluence of so many factors simultaneously? Resolving these issues is beyond the scope of traditional, linear, closed-system methods. Viewing food system sustainability from a dynamic systems perspective makes it possible to examine non-linear, complex, and reciprocally causal processes more explicitly. In the following section, we build on system thinking to identify the main variables to formalize and operationalize the abstract and multidimensional concept of sustainable food systems.

## Formalizing the Food System as a Dynamic System

### Defining Dynamic Systems

The term “dynamic system”—or “dynamical system”—refers to a set of interacting elements that change over time. The first assumption of the dynamic approach is that evolving systems are complex, i.e., composed of many individual elements embedded within, and open to, a complex environment. These elements function together as a collective unit, producing outputs in relation to inputs through processes endogenous to the system. Changes in one variable will impact all other variables of the system, with possible lagged and multi-scale effects. Outcomes thus emerge from the complex interactions among system elements, potentially including natural as well as human components, and are not just the product of external causes.

The field of dynamic systems is vast. From initial work in cybernetics (Wiener 1948; Ashby 1956) and system theory (Kalman et al. 1962; Bertalanffy 1968), system thinking grows directly from advances in physics and mathematics. Psychology also uses system-based approaches to explore human behavioral patterns. The more technical term “dynamic system modeling” refers to a class of mathematical equations that describe time-based systems with particular properties. Systems can be classified in different ways. System models can be either continuous or discrete. They can be linear or non-linear, and time invariant or time variant. A system can be static if its output depends only on its present input. On the contrary, a dynamic system requires past input to determine the system output.

A dynamic systems approach begins with defining problems dynamically, proceeds through modeling stages, then builds confidence in the model and its policy implications. As highlighted in the previous section, change is key to sustainability. Sustainability is about maintaining and/or enhancing essential functions or outcomes over time, taking into account environmental, social, and economic constraints and assets. Food system sustainability can be viewed as the ex ante assessment of potential change in its functioning, given external conditions and internal dialectic. More precisely, it aims at capturing (and protecting) the properties of the system crucial to supporting life, including food security that is the first reason for being of food systems (Haddad 2013). This requires examining how the multicausality of dynamic processes within complex systems such as the food system could help understand changes over time towards food security.

### A Mathematical Representation

**Fig. 2:**
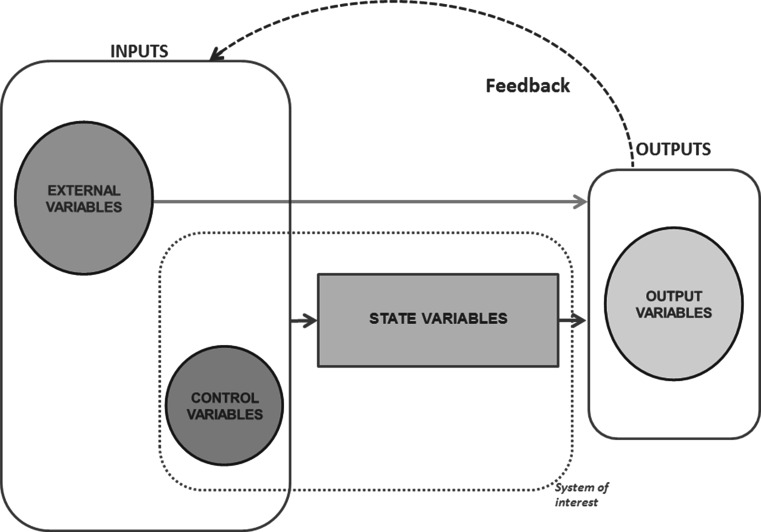
Basic representation of a dynamic system (adapted from Rastoin and Ghersi 2010)

Modeling dynamic systems is about representing mathematically the dynamics between the inputs and outputs of the system of interest. Figure 2 shows a simplified graphical representation of a dynamic system. Specifically, it depicts a closed-loop dynamic system with feedback from outputs to inputs. A “controller” can monitor the output *y* of the system by adjusting control variables *u* to achieve a specified response. When modeling input–output systems, in addition to an observed set of variables internal to the system that can be levers of action, external drivers *e* can enter the model as inputs (Ionescu et al. 2009). If considered as exposed to external influences, the system is said to be non-autonomous (Stankovski 2014). Dynamic systems can also be perturbed by unobserved forces or noise. For the sake of simplicity, the presentation below is made under deterministic assumptions. For approaches motivated by stochastic models, see Aström (2012) and references therein.

Not all variables that appear in a model are of interest. The behaviors are usually captured by defining appropriate outputs. We choose outputs in order to describe those quantities that get focus. In this paper, food and nutrition security is considered as the principal outcome of food systems, as it should be its main reason for being (Burlingame and Dernini 2011; Haddad 2013; Allen et al. 2014). These outcomes are also determined by decisions and actions taken along the activities of the food system, but also by global socio-economic, political, and environmental drivers through their impacts on the food system (Ingram et al. 2010). Such drivers might also impact food security directly.

The state of the system at a given time is the extra piece of information needed, so that given the input trajectory, it is possible to determine the behavior of the system over time. We call *x* the state variables of the system. They provide the minimum amount of information that describes the system at any given time *t*. A mathematical description of the system in terms of a minimum set of variables *x*, together with knowledge of those variables at an initial time *t*_0_ and the system inputs for time *t*, is deemed sufficient to predict the future system states and outputs for all time *t*.

A set of equations can be used to describe the behavior of the system. Output functions are commonly used to characterize the input–output relationships. The dynamics of the system are usually represented using differential or difference equations (with time as the independent variable). These equations, known as the transition functions, are formulated in state-space form that has a certain matrix structure.

The output equations are commonly written as^4^

1 $$y_{t} = h\left( {x_{t} ,u_{t} ,e_{t} } \right),$$

where $h$ is a vector function with *n* components for the *n* outputs *y* of interest. All variables typically vary with time *t*.

Transition functions map the state of the model today into the state tomorrow. In vector notation, the set of differential equations may be written as

2 $$\dot{x} = \frac{dx}{dt} = f\left( {x_{t} ,u_{t} ,e_{t} } \right),$$

where *f* is any vector function. The system state at any instant *t* may be interpreted as a point in an *m*-dimensional state-space,^5^ and the dynamic state response *x*_*t*_ can be interpreted as a trajectory traced out in the state-space (Rowell 2002).

Further two Eqs. (3 and 4) can be added to the usual differential equation to map the feedback to inputs (Ionescu et al. 2009). The problem of parameter estimation pertains to the identification of data and determination of numerical values of the elements of these matrices.

3 $$\dot{e} = \frac{de}{dt} = g\left( {x_{t} ,u_{t} ,e_{t} } \right)$$

4 $$\dot{u} = \frac{du}{dt} = \emptyset \left( {x_{t} ,e_{t} } \right)$$

### Categorizing Variables, Constructing a Composite Indicator

As explained in “Identifying the Fundamental Sustainability Properties of the Food System” section, we are looking for the essential variables describing a system and the variables we can act upon to redirect food systems toward regarded objectives. In the language of dynamic systems, we are looking for *x* and *u*, the state and control variables, respectively. These are the essential features of the system that determine the trajectory of the system and characterize its sustainability. A system can be understood by the response pattern following a perturbation; perturbation reveals the nature of the system. To capture something of the internal dialectic of a system, we suggest fixing some crucial external variables, or drivers of changes *e*, and observing how these affect our system outcome of interest: food and nutrition security.

The concepts from the already existing vulnerability/resilience framework can then allow us to clarify what we would like to proxy; literally, vulnerability is the propensity or predisposition of a social-ecological system to be adversely affected by a change. Some global processes are significant drivers of change. There is high confidence that these include population growth, rapid and inappropriate urban development, international financial pressures, increases in socio-economic inequalities, and trends and failures in governance.

As presented above, vulnerability/resilience is made up of three essential components: exposure, sensitivity, and resilience. Thus, vulnerability *V* can be regarded as a function of the components’ recovery potential (*RP*) and potential impacts (*PI*), which in turn are expressed by exposure (*E*) and sensitivity (*S*).

5 $$V = f\left( {PI, RP} \right), {\text{with}} \, PI = f\left( {E,S} \right)$$

The vulnerability/resilience framework can help articulate the different elements of the system of interest, i.e., categorize variables with regard to others, and construct a composite indicator. This causal modeling approach is critical in the absence of statistical application able to reveal the structure of the data through procedures such as Principal Component Analysis (PCA). See Prosperi et al. (2014) for a proposition of composite indicator. Second, the vulnerability/resilience framework allows articulation of the different scales at which food systems are operating or embedded in.

**Fig. 3:**
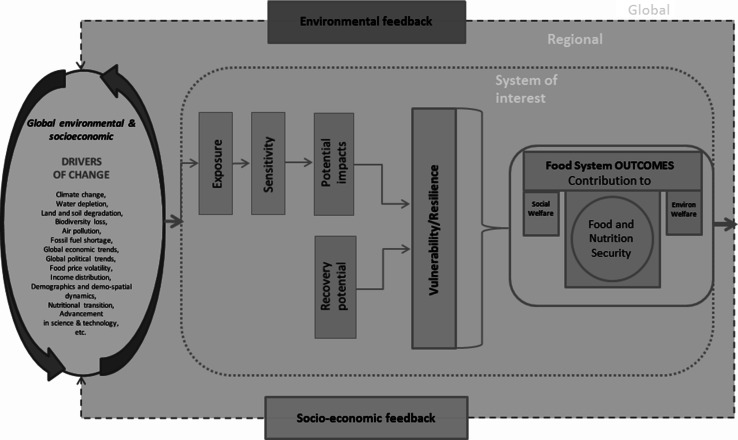
A Sustainable food system framework (adapted from Turner et al. 2003; Ericksen 2008; Ingram 2011)

Building on the GECAFS food systems approach (Ericksen 2008; Ingram 2011), coupled with Turner et al.’s (2003) conceptualization of vulnerability, we suggest the framework represented in Fig. 3 to model food systems’ dynamics. Dynamic systems contain mainly two types of variables: endogenous and exogenous variables. Endogenous variables are the elements that are interactive within the boundaries of the system of interest. In the case at hand, these variables are defined at the national or sub-national level. On the contrary, exogenous variables are factors that are not enclosed by the system boundary but influence the system. Exogenous variables are, conversely, not *directly* influenced by variables enclosed within the system. Outcomes from the food system activities may however contribute to these external drivers, but geographically specified food systems are assumed *driver*-*takers.*^6^ In our specific case, these external drivers of change are at the broader regional level or global scale. The three components of vulnerability—exposure, sensitivity, and resilience—are the intrinsic features of the system that mediate the impact of the drivers of change on the food system’s outcomes. These can be either state or control variables.

In this section, we specifically consider the large body of research on dynamic systems, and aim at applying this modeling approach to the assessment of food system sustainability. To assess the sustainability of the food system, we need to understand what might affect its processes, to what extent the drivers of change impact the food system’s outcomes, and how actors respond to these pressures. Answering the question that was first posed by Carpenter et al. (2001)—“the resilience of what to what” or, in a similar vein, “vulnerability of what to what”—can provide useful guidance.

## Application: Addressing Context-Specific Issues

### A Stepwise Approach

Schroeter et al. (2005) developed an eight-step methodological process to conduct vulnerability assessments. Following Schroeter et al., we propose a similarly structured and systematic method to apply the conceptual elements described in the above sections. These steps are preliminary to the identification of appropriate statistical variables, data application, and scenario analysis. They involve proceeding in four stages: 1. defining a study area and scale of analysis; 2. identifying essential drivers of change; 3. identifying essential food systems’ outcomes; and 4. developing a causal model by selecting essential interactions, drivers, and outcomes, and examining respective systems’ exposure, sensitivity, and recovery potential.

Sustainability is usually conceived in place-specific terms. In the proposed framework, exposure to risks is dependent on the geographical context, and sensitivity and adaptive capacity are shaped by social and institutional factors (Eakin 2010). The first step includes choosing a scale of analysis and drawing artificial boundaries around the coupled human–environment system of interest. Every system incorporates some sub-systems, which are themselves based on components, which are in fact sub-systems, etc. Two points are crucial to consider when defining the system level and spatial scale of analysis: (i) who are the intended users of the measurement set and (ii) what is the degree of granularity of the food system’s outcomes to be addressed.

This work is part of the project “Advancing through sustainable diets” that focuses on France and Spain. Given that the assessment is targeting policy-makers as main users, we opted for analysis at the population scale rather than the individual scale. It has thus been decided that the final level of analysis will be national or sub-national (“Comunidad autónoma” in Spain, “Région” in France and “Regione” in Italy). To draw the geographical boundaries, it has then been argued that the entities had to be subjected to similar types of food system concerns and exposed to similar types of drivers of change or factors of risk. Italy has thus been added to France and Spain as a possible study zone, on the grounds that the three countries share similar food and nutrition security issues.

The northern coastal area of the western Mediterranean basin is commonly referred to as the “Latin Arc.” It includes the coastal regions from Andalusia to Sicily. It is considered a homogeneous geographical entity closely related to certain summary representations of the European territory, at the regional level, proposed by geographers and urban scholars (Voiron-Caniccio 1994; Daviet 1994; Barrio 2004; Vanolo 2007; Camagni and Capello 2011). It is also recognized as a consistent territory by institutions and local stakeholders for transregional policy and cooperation programs (e.g., Western Mediterranean and Latin Alps, INTERREG II C Programme, EU) (Benoit and Comeau 2005), sharing common cultural, institutional, socio-economic, and biogeographical determinants.

As mentioned previously, the spatial scale at which the system is defined drives the identification of the external variables likely to affect the system. Sub-global/regional level is a natural level to specify these external drivers of change in SES studies. The Mediterranean basin has been identified as one of the most prominent “hotspots” in future climate change projections (Giorgi 2006), but also in terms of environmental unsustainability due to intense human activity and agricultural exploitation (Salvati 2014). It has also been recognized as one of the first 25 Global Biodiversity hotspots in the world (Myers et al. 2000).

### Identifying Global and Regional Drivers of Change Affecting the Food System Outcomes

The second and third steps are crucial in applying the conceptual framework. It involves answering the question “vulnerability/resilience of what to what.” It also requires simultaneously identifying the main drivers of change as the food system-specific issues of concern that the drivers are likely to affect (Schröter et al. 2005). Several global and regional drivers of change affect the structure and processes of food systems, putting context-specific food and nutrition security outcomes at risk. Based on an extensive literature review and discussions conducted throughout two focus group sessions made up of seven experts, four critical food and nutrition security issues and four drivers of change were identified at a sub-regional level. An exhaustive and rigorous literature review specific to the Mediterranean region highlighted existing urgent issues and crucial drivers of change (CIHEAM 2012; SCAR 2008; PARME 2011). The selected four main drivers of change are the following:

#### Water Depletion

Water depletion is “a use or removal of water from a water basin that renders it unavailable for further use” (Molden 1997). The Mediterranean region is greatly concerned by water stress and scarcity (PARME 2011; FAO 2011). The Western and Central Mediterranean areas are particularly subject to increasing water needs for domestic use and tourist and agricultural activities (Sousa et al. 2011). Water demand has doubled over 50 years in Mediterranean countries (UNEP/Blue Plan 2006). The food system production and consumption patterns are increasingly water demanding. Irrigated agriculture only accounts for 70 % of the consumption of freshwater resources globally (OECD 2013). In EU-27, the majority of irrigated areas are concentrated in the Mediterranean region; 75 % of the total area equipped for irrigation in EU-27 is located in France, Spain, Italy, Portugal, and Greece (Wriedt et al. 2008).

Water availability is closely related to climate change trends altering precipitation patterns and rainwater (Freibauer et al. 2011). Increase in the concentration of agrochemicals and soil nutrients, and a number of water pollutions have also been observed, impacting the quality of water and further contributing to water scarcity (Bates et al. 2008).

#### Biodiversity Loss

Biodiversity^7^ loss is defined as “the long-term or permanent qualitative or quantitative reduction in components of biodiversity and their potential to provide goods and services, to be measured at global, regional and national levels” (CBD 2004). Biodiversity is globally at risk, with 20930 species and ecological communities known to be threatened (IUCN 2013). The Mediterranean region has been, in particular, cataloged as one of the 25 biodiversity hotspots of the planet with an exceptional diversity of endemic species within ecosystems that are at great risk, with 19 % of the species threatened by extinction (IUCN 2008).

Biodiversity loss is simultaneously generated by climate change, environment depletion, and water stress. It is strongly related to modern food production and consumption patterns (Altieri 2000) that have become more intensive and homogenizing. The loss of agrobiodiversity is interlinked also with a number of causal factors including habitat depletion, change in land use and management, and GHG emissions among others (Tilman et al. 2002; Frison et al. 2011).

#### Food Price Volatility

Food price volatility refers to large and atypical^8^ “variations in agricultural prices over time” (FAO 2011). Food prices increased sharply in 2008, with the FAO food price index breaking the threshold of 200^9^ for the first time (SCAR 2008). The Mediterranean region is a particularly vulnerable region with regard to price volatility due, in particular, to several factors including its cereal dependence, nutrition transition, population growth, urbanization, and climate change (Padilla et al. 2005).

Climate change impacts, changing trade patterns, new dietary trends, and growing demand for biofuels are often quoted as being among the causes of food price volatility. The rising demand for food and fuel originating from consumption and industrial purposes is engendered by both population growth and changes in food consumption patterns (Brown 2008). Furthermore, speculation on commodity markets and reduction of food stocks are also crucial determinants of price variations (Robles et al. 2009).

#### Changing Food Consumption Patterns

Changing food consumption^10^ patterns refers to the changing structure of global food consumption, related to changing dominant values, attitudes, and behaviors (Kearney 2010a, b). Globally, food consumption patterns are changing both in terms of total amount and composition. Worldwide consumers have switched from considering animal protein a luxury food item to considering it a regular part of the diet (Meade et al. 2014).

Food choices are deeply embedded in social norms. Individual food consumption patterns—i.e., diets—are the result of changes in culture, social values, and representations attached to food consumption. The global changes in food consumption patterns—some talk about a “westernization” of food consumption patterns (Drewnowski and Popkin 1997)—are largely driven by demographic factors and income growth and are related to changes in dominant values and lifestyle influenced by globalization, urbanization, changes in occupational status and employment distribution, and more effective dissemination of information (Meade 2012).

### Identifying Food and Nutrition Security Issues

It is important at this point to formalize the hypotheses to be explored. The “what is vulnerable” is identified by the functions performed by the ecological and social service delivering entity composed of a number of actors, activities, and processes. The system will be considered vulnerable if negative food system outcomes emerge. Food, or more precisely, feeding population, is agriculture and food systems’ main reason for being (Haddad 2013). Human nutrition should be considered one of the most fundamental ecosystem services, or alternatively as dependent on several ecosystem services, including provisioning, regulating, and supporting, and cultural services (Deckelbaum et al. 2006).

Food security, defined as the situation that exists when all people, at all times, have physical and economic access to sufficient, safe, and nutritious food to meet their dietary needs and food preferences for an active and healthy life (FAO 1996), is a policy issue of importance in just about every country. It can be considered the principal outcome of food systems. It is also important to remember that food security is not just about the amount of food but also depends on the nutritional quality, safety, and cultural appropriateness of foods (Liverman and Kapadia 2010). Investigating the influence of socio-economic and environmental drivers on food and nutrition security, with regard to some essential food system characteristics, provides an approach to think the causal mechanisms that can lead to unsustainability. As mentioned above, four food and nutrition security issues have been identified as crucial for the Latin Arc countries.

#### Nutritional Quality of the Food Supply

The Nutritional quality of food supply refers to the nutritional composition of the food products on the market (Oquali, INRA/ANES). The improvement of the nutritional quality of the food supply is one of the eight specific actions defined by the WHO European Action Plan for Food and Nutrition Policy 2007–2012.^11^ A balanced diet is achieved through personal habits but also requires that the foods on offer to consumers have a satisfactory nutritional composition. In France, a food quality observatory (Oqali) was set up to monitor the quality of the food supply. Increasing availability and consumption of nutrient-poor and energy-dense foods and beverages leads to enhancement of human health problems, including obesity and non-communicable chronic disease.

#### Affordability of Food

According to Ingram (2011), affordability of food is “the purchasing power of households or communities relative to the price of food.” It refers to the “economic access” to food (Foran et al. 2014). Affordability is about food being available at prices that people can afford to pay and, in particular, whether low-income consumers can afford to buy enough nutritious food to meet basic needs (Barling et al. 2010). The determinants of food affordability include pricing policies and mechanisms, seasonal and geographical variations in price, local prices relative to external prices, the form in which households are paid, and income and wealth levels (Ericksen et al. 2009). Food affordability and food prices are important determinants of food choices (Lee et al. 2013).

#### Dietary Energy Balance

Dietary energy balance refers to the balance between caloric intake and energy expenditure (Patel et al. 2004). Excessive fat accumulation is acknowledged to be a risk factor for various health problems, including cardiovascular disease (CVD), diabetes, cancers, and osteoarthritis (WHO 2014). Obesity has become a significant public health issue in high- and medium income countries, with 500 million adults obese worldwide and more than 1 billion projected by 2030 if no major effort is made (Kelly et al. 2008; Finucane et al. 2011). Body weight results from the integrated effects of food consumption, physical activity, and genetics. Environmental, social, and behavioral factors interact to determine energy intake and expenditure. Sedentary lifestyles, heavy marketing of both energy-dense foods and fast food outlets, adverse social and economic conditions, and the consumption of high-sugar drinks, among others are driving a dietary energy imbalance, with higher calorific intake and lower energy expenditure (WHO 2000; Swinburn et al. 2004).

#### Satisfaction of Cultural Food Preferences

Cultural food preferences are environmental factors related to social background that contribute to food choices and intake. It is now acknowledged that honoring ethnic and cultural food preferences, compatible with nutritional requirements, is essential for food acceptance and general wellbeing. Social and cultural norms have a crucial role in diet (Sobal et al. 1998). Food preferences, socially or culturally determined, are now recognized as a key consideration in food security, as highlighted already in the 1996 definition of food security. Assessing cultural issues surrounding food preferences may also help improve dietary adherence to recommendations.

### A Causal Model

In the fourth step, a causal model is developed, formalizing the dynamics of exposure, sensitivity, and resilience. The four drivers of change and four food security issues presented above are matched to explore their possible causal relationships. The proposed framework aims at identifying the food system characteristics that make the food system capable of sustaining food and nutrition security outcomes. This can serve to identify warning signals, although the drivers and outcomes of interest will have to be evaluated as well. Results are presented in Table 1.

**Table 1:** Interaction drivers of change/FNS issues

**Drivers of change**		References
* Food and nutrition security issues*		
**Water depletion**		
* Nutritional quality of food supply*		
Potential impact	Contributing to the decrease of production and productivity of sufficient and nutritious foods Engendering low dilution capacity and consequent contamination of agrofood products Impacting the availability of quality foods for poor consumers through higher cost of water	(Bates 2008; SCAR 2008; Brown 2008; Ericksen et al. 2010; Wood et al. 2010; PARME 2011; Dangour et al. 2012; Johnson et al. 2014)
Recovery potential	Fostering water productivity and efficiency to guarantee adequate nutritional values of foods Contrasting water scarcity through agrobiodiversity richness Enhancing adaptation through food import from water-rich countries Reuse wastewater safely for use as water sources Focusing on human capacities and institutional framework	(Chapagain et al. 2006; SCAR 2011; Prosperi et al. 2014; UNWATER 2014)
* Affordability of food*		
Potential impact	Altering productivity, prices, and trade, and then food availability and affordability Increasing water prices leads to higher costs of agrofood production and to a decrease in food affordability	(Ingram and Kapadia 2010; Wood et al. 2010; SCAR 2011)
Recovery potential	Encouraging drought-resistant crops utilization Fostering food import from water-rich countries Improving irrigation efficiency Promoting waste water treatments	(Hellegers et al. 2008; Waughray 2011; Yang and Zehnder 2008; Prosperi et al. 2014)
**Biodiversity loss**		
* Nutritional quality of food supply*		
Potential impact	Shifting to ecologically simplified systems based on cereals contributes to poorly diversified diets Hampering food systems responses against climate change, with consequent impact on productivity Increasing the dependency on global varieties on external inputs	(Randall et al. 1985; Torheim et al. 2004; Pelletier and Frongillo 2003; Frison et al. 2006; Roche et al. 2008; SCAR 2008; Arimond et al. 2010; Remans et al. 2011; Dangour et al. 2012; SCAR 2011; Allen et al. 2014; Johnson et al. 2014)
Recovery potential	Promoting agrobiodiverse systems for ecosystem services, food security benefits (nutritional value of foods), the viability of agricultural systems, and long-term productivity Fostering organic farming	(Thrupp 2000; Reidsma and Ewert 2008; Eakin 2010)
* Satisfaction of cultural food preferences*		
Potential impact	Putting at risk cultural traditions and preferences, linked to regional varieties and diets Homogenizing food production Contributing to reduce the enormous amount of information, on nutritional and health benefits of the foods that shape the food cultural preferences of people Decreasing food biodiversity could result in the loss of unique and traditional foods	(Kuhnlein et al. 2009; Kearney 2010a, b; Liverman and Kapadia 2010; SCAR 2011; Jacques and Jacques 2012)
Recovery potential	Knowing how to prepare a more varied diet can influence the consumption of different food products Providing more varied and tasteful diets Enhancing and keeping traditional food cultures	(Termote et al. 2010; Johnson et al. 2014; Khoury et al. 2014)
**Food price volatility**		
* Nutritional quality of food supply*		
Potential impact	Impacting food production and consumption Altering food supply towards disadvantaged groups Leading to profound changes in the composition and availability of food supplies Hampering the present agrofood system supply, strongly interlinked with fossil fuel system	(DEFRA 2008; SCAR 2008; Friel and Lichacz 2010; WHO 2014)
Recovery potential	Enhancing dietary diversity for avoiding dependency on few groups of foods Fostering local provisioning and production, less involved in price variations	(Pinstrup-Andersen 2013)
* Affordability of food*		
Potential impact	Impacting household incomes and purchasing power Affecting agrofood productivity, and therefore food affordability and availability Exacerbating economic shocks for the poor, who depend on wages and the rest of the economy Shifting purchasing strategies to lower quality products	(Ingram 2008; SCAR 2008; UK Cabinet Office 2008; Wood et al. 2010; HLPE 2011; SCAR 2011; Lee et al. 2013; Regmi and Meade 2013)
Recovery potential	Fostering food industry’s focus on consumers and their need for “affordable food of high quality and diversity” Shifting towards cheaper or locally available foods, meeting the same caloric and nutritional requirements Implement food policies for diversifying supply sources through different strategies (subsidies, food stamps) Promoting diversity in food consumption patterns	(European Technology Platform 2008; Brunori and Guarino 2010; Prosperi et al. 2014)
**Changes in food consumption patterns**		
* Nutritional quality of food supply*		
Potential impact	Influencing food industry production patterns, overall food security, and nutritional characteristics of diets Shifting the demand towards cereals, simple sugars, animal products, and highly processed foods	(European Technology Platform 2008; Brunori and Guarino 2010; SCAR 2011; UNEP 2012; Prosperi et al. 2014; WHO 2014)
Recovery potential	Improving the understanding of the determinants of consumer choices Empowering consumers’ choice for healthy and safe provided food Engendering consumption patterns cognizant of the impact of food choice on health	(SCAR 2011; Khoury et al. 2014; Allen et al. 2014)
* Dietary energy balance*		
Potential impact	Increasing consumption of fats, sugars, sweeteners, animal products, highly processed foods, and fast foods and vending machine products Decreasing consumption in plant proteins and of home-prepared foods Strengthening “obesogenic” environments with little energy expenditure and sedentary lifestyles Altering frequency and the amounts consumed of foods Decreasing dietary diversity	(Swinburn et al. 1999; UNSSCN 2004; Popkin 2002; Nielsen and Popkin 2004; Garrett and Ruel 2005; Ley et al. 2006; SCAR 2008; Ericksen et al. 2010; Friel and Lichacz 2010; Liverman and Kapadia 2010; PARME 2011; SCAR 2011; Lozupone et al. 2012; UNEP 2012; Yatsunenko et al. 2012; Johnson et al. 2014)
Recovery potential	Fostering public awareness for healthier diets through campaigns and community movements Enhancing cultural knowledge on preparing varied diets and on nutritional and health benefits of the foods Promoting weight loss and metabolic health through appropriate changes in the gut microbiota Supporting guidelines on dietary strategies to counteract overweight and obesity	(Barling et al. 2010; Kuhnlein et al. 2009; Obersteiner et al. 2010; Termote et al. 2010; Cardoso et al. 2013; Freeland-Graves and Nitzke 2013; Lopez-Legarrea et al. 2014)

These sets of characteristics are indicating how changes in water, biodiversity, food prices, and food consumption patterns are transmitted through the food system, including the sequencing of events and the scale of interactions; how the food system is sensitive to these changes; and the adaptive capacity of the food system. This could lead to subsequent work to identify thresholds of change and to quantitatively model the interactions among stressors, attributes, and outcomes, to improve the general understanding of food system sustainability. It more importantly presents the elements that need to be assessed, i.e., the attributes for which indicators can be used to measure and monitor.

## Discussion

### Why Vulnerability and Resilience to Assess Sustainability?

In this paper, we propose the analysis and assessment of the sustainability of food systems using the concepts of vulnerability and resilience. First, vulnerability is not the simple flip side of resilience. Following Turner et al. (2003), we argue that articulating the two—overlapping—concepts provides a more comprehensive framework to capture the features of complex systems, such as food systems, that perpetually evolve and re-organize into unexpected new configurations. The identification of the elements within the system, and assessment of their sensitivity to change, in addition to the capacity of the system to cope, adapt, and transform to these changes, is considered key to understanding dynamic systems. Resilience and vulnerability are relatively new, yet are now fundamental concepts in the contemporary language of sustainability sciences. The links between vulnerability and sustainability have been discussed against the backdrop of a long-standing dispute about the relationship between sustainability and resilience. Resilience is commonly accepted as at least a crucial dimension of sustainability. Some argue that resilience of a system constitutes a necessary but not sufficient condition for sustainability (Derissen et al. 2011). The question remains, however, of how the concepts of vulnerability and resilience square with the definition of sustainability.

Sustainability is a *normative* concept that provides a broad framework to guide actions. It requires defining specific goals—and their monitoring measures—that need to be agreed upon and acknowledged by all stakeholders (Anderies et al. 2013). On the contrary, resilience and vulnerability, as *descriptive* concepts, characterize the dynamic properties of a system and can thus help define these societal goals. Sustainability and vulnerability/resilience can thus be understood as distinct concepts operating at different levels, the latter concepts providing the elements to inform the decision process intrinsic to the former concept.

Although the concepts of “vulnerability” and “resilience” have entered the food policy discourse, the influence of SES thinking on policy-maker agendas has otherwise been rather limited (Foran et al. 2014). SES frameworks emphasize complexity and systemic interactions. Applications of these frameworks tend thus to focus on problem identification and improving system understanding (Nadasdy 2007). As mentioned earlier, food systems are systems of variables connected to each other through causal pathways, which are further complicated by operating on different geographical or time scales. These connections need to be grasped and theorized. Vulnerability and resilience can be useful approaches to capture these relationships. One key conceptual element of vulnerability/resilience models is a clear distinction between causal events and outcomes (Dilley and Boudreau 2001). It frames a “causal factor approach” that describes the interactions leading to the final outcomes. Exposure, sensitivity, and resilience provide the concepts to identify the system’s properties that shape causal pathways towards food system outcomes.

Systems behave in a circular organization forming feedback loops. The proposed fragmentation in specific vulnerabilities and resilience factors—through the intersections of different drivers and issues—can induce a certain degree of linearity in causality. Vulnerability and resilience answer questions about mechanisms that operate to produce outcomes under certain specific conditions. As such, these two properties provide policy-makers with a model of highly formalized predictions of the effects of a limited set of variables (Epstein et al. 2013) that can be tested recursively and provide insights into possible feedback. Modelers are generally faced with the dilemma of how comprehensive a model to build: “one with many variables that ends up as a qualitative description, or one with a few key variables that acts quantitatively but lacks comprehensiveness” (Fraser et al. 2005). It must also be borne in mind that sustainability as a forward-looking concept requires apprehending the conditions and determinants needed to maintain systems’ functions *over time*. Focusing on a number of external forces and highlighting systemic internal dialectic, the vulnerability/resilience model allows a dynamic analysis of some specific issues of the food systems and provides direction for policy-makers.

### Why these Specific Issues and Drivers?

Building on Schroeter et al. (2005), two of the four sub-steps proposed to resolve the complexity that arises when integrating social and ecological approaches imply specifying food systems’ outcomes and external drivers. It requires first clarifying the principal outcomes or functions of a food system, in particular the issues at risk. Food systems serve several purposes and have several outcomes. Outcomes are susceptible to being evaluated and ranked differently by different stakeholders, and at different levels. The proponents of the “Sustainable Diet” agenda highlight the food and nutrition security objectives of the food systems selected here as the end-point of the analysis (FAO/Bioversity 2012). As mentioned above, following a review and after discussion in two focus groups, four food and nutrition security concerns have been retained, judged crucial to the context at hand. Other issues, however, have been debated such as “food safety” or “dietary quality.” Other food systems’ outcomes than food and nutrition security issues could also have been considered, such as environmental and socio-economic outcomes related to employment or equity. Food systems are responsible for diverse environmental, economic, and social outcomes, and introducing these may have been more in line with the generally accepted understanding of sustainability. The articulation between food systems’ defining elements and their resulting outcomes, the former contributing to predict the latter, could be expanded to other dimensions to further the modeling approach. Sustainability can hardly be modeled parsimoniously, raising then questions in terms of feasibility of the modeling.

The second step is to understand what and how global or regional changes, either socio-economic or environmental, might be transmitted through the activities to impact the outcomes, because food systems’ complexity means that impacts may not always be felt directly. Experts invited to the focus groups mentioned other important drivers of change, such as “climate change” or “technological innovation.” They also considered whether or not the model completely captures the internal drivers that are intrinsic to the system. Drivers are interacting with each other. Climate change and biodiversity loss for example are closely related and highly susceptible to reciprocal influence. This interdependence raises some technical modeling concerns such as named variables acting as possible proxy for other variables associated with them. A distinct effort was thus made to select priority drivers and to exclude those drivers exhibiting direct reciprocal influence.

The analysis of the connections linking global and regional drivers of change with context-specific food system outcomes could be also carried out through polycentric governance approaches. Considering a polycentric order as “one where many elements are capable of making mutual adjustments for ordering their relationships with one another within a general system of rules where each element acts with independence of other elements” (Ostrom 1999, p. 57), actors can use local knowledge and participate in iterative and reflexive learning processes where other stakeholders are involved. Since polycentric systems imply mutual monitoring and learning, knowledge, innovation, adaptation, credibility, and cooperation between stakeholders can improve over time and enhance the sustainability of the system at multiple scales (Toonen 2010). In particular, single context-specific governance units could be deemed as key components able to respond—to the impacts from global and regional changes—with diverse and multiple scale interventions and solving strategies for *collective*-*action problems* (Ostrom 2010). From this polycentric governance perspective, then, it might be possible to define alternative scales of analysis and draw different artificial boundaries within the food system, in its collective units and sub-systems, and the degree of granularity of the food system’s outcomes. Thus, a polycentricity-based framework of sustainability might imply to consider alternative social and institutional factors in order to describe exposure, sensitivity, and adaptive capacity.

Finally, some analytical clarity and direction are essential to convince policy-makers and thus guarantee impact of policy. It is more highly desired to develop interventions that treat the underlying causes, rather than the symptoms of unsustainability of food systems. The concepts of vulnerability and resilience bring food security into consideration in a new and alternative way. Change is occurring and investigation of the sources of adaptive capacity of the system is crucial.

## Conclusion

Developing policy to ensure sustainable food security is a tremendous challenge that requires a comprehensive and integrated analytical approach. Multiple factors influence the course of human–environment interactions, which are further complicated by the presence of co-evolving causal forces. Understanding these dynamics requires viewing the food system as a whole. Social-ecological system approaches allow us to move away from looking at isolated events and their causes, and to begin looking at systems made up of interacting parts. A vulnerability and resilience approach is suggested here as a possible framework to capture the food system as a whole, think prospectively and identify the system elements that policy can leverage. The distinction in three components, namely exposure, sensitivity, and resilience, provides the elements of a model that specify which attributes need to be measured and how to structure the different indicators in a coherent framework for improved decision making and policies.

The concepts of vulnerability and resilience impose a system thinking approach based on the interdependencies between drivers, system activities and properties, outcomes, and feedback loops. Vulnerability and resilience of food systems can have multiple sources, and these sources may interact to generate unexpected responses (SCAR 2008). As sustainability and food security become increasingly central, vulnerability/resilience will be among the principles that will drive the reformulation of research, as well as policies (Brunori and Guarino 2010). As powerful tools capable of monitoring global change, vulnerability/resilience assessments represent a new research frontier; however, more theoretical and empirical research is needed to measure and assess the interplay between human and environment systems, between causal factors and consequences. Furthermore, the development of appropriate tools is required for monitoring, forecasting, and integration in policy support measures.

## Appendix

See Table 1.

## Footnotes

1 This work is part of the project “Advancing through sustainable diets” that has a focus on France and Spain.

2 The IPCC definition mentions specifically “people; livelihoods; environmental services and resources; infrastructure; or economic, social, or cultural assets” (2012).

3 The IPCC Third Assessment Report describes vulnerability as “The degree to which a system is susceptible to, or unable to cope with, adverse effects of climate change, including climate variability and extremes. Vulnerability is a function of the character, magnitude, and rate of climate variation to which a system is exposed, its sensitivity, and its adaptive capacity” (IPCC 2001, p. 995).

4 The notation below is a vector notation, which allows us to represent the system in a compact form.

5 With *m* variables determining the state of the system.

6 In the same way as consumers or producers are considered price-takers, in perfect competition, although price is collectively defined when overall demand and supply meet.

7 Biodiversity is defined as the existence of species, genetic, and ecosystem diversity in an area (Swingland 2000).

8 See FAO report *Price Volatility in Food and Agricultural Markets: Policy Responses* (Appendix, 2011) for a more technical definitions of price volatility.

9 Base 100: 1998–2000.

10 This social driver is proposed as one regime driver by the SCAR 2nd Foresight exercise report (2009), closely linked to the other social global driver “changing dominant values,” and is exactly phrased “Consumption quantities and patterns,” referring literally to “food consumption patterns” and “nutritional transition.”

11 The WHO European Action Plan for Food and Nutrition Policy 2007–2012 defines eight specific actions for “ensuring a safe, healthy and sustainable food supply: improve the availability and affordability of fruit and vegetables; promote the reformulation of mainstream food products; promote appropriate micronutrient fortification of staple food items and develop complementary foods with adequate micronutrient content; improve the nutritional quality of the food supply and food safety in public institutions; ensure that the commercial provision of food products is aligned with food-based dietary guidelines; explore the use of economic tools (taxes, subsidies); establish targeted programs for the protection of vulnerable and low socioeconomic groups; establish intersectoral food safety systems with a farm-to-fork approach and in accordance with the Codex Alimentarius risk analysis framework.”
